# A Capacity-Achieving Feedback Scheme of the Gaussian Multiple-Access Channel with Degraded Message Sets

**DOI:** 10.3390/e23060756

**Published:** 2021-06-16

**Authors:** Haoheng Yuan, Bin Dai

**Affiliations:** 1 School of Information Science and Technology, Southwest Jiaotong University, Chengdu 611756, China; yhhcd@my.swjtu.edu.cn; 2 The State Key Laboratory of Integrated Services Networks, Xidian University, Xi’an 710071, China

**Keywords:** Gaussian multiple-access channel, capacity region, feedback, SK scheme

## Abstract

The classical Schalkwijk–Kailath (SK) scheme for the point-to-point white Gaussian channel with noiseless feedback plays an important role in information theory due to the fact that it is capacity-achieving and the complexity of its encoding–decoding procedure is extremely low. In recent years, it has been shown that an extended SK feedback scheme also achieves the capacity region of the two-user Gaussian multiple-access channel with noiseless feedback (GMAC-NF), where two independent messages are, respectively, encoded by two intended transmitters. However, for the two-user GMAC-NF with degraded message sets (one common message for both users and one private message for an intended user), the capacity-achieving feedback scheme remains open. In this paper, we propose a novel two-step SK-type feedback scheme for the two-user GMAC-NF with degraded message sets and show that this scheme is capacity-achieving.

## 1. Introduction

Shannon showed that feedback does not increase the capacity of the discrete memoryless channel (DMC) [1]. Later, Schalkwijk and Kailath [2] proposed a feedback coding scheme called the SK scheme and showed that this scheme is capacity-achieving and greatly improves the encoding–decoding performance of the point-to-point white Gaussian channel. According to the landmark paper [2], Ozarow [3] extended the classic SK scheme [2] to a multiple-access situation; namely, the two-user Gaussian multiple-access channel (GMAC) with noiseless feedback (GMAC-NF), where two independent messages are, respectively, encoded by two intended transmitters. Similar to [2], Ozarow showed that this extended scheme is also capacity-achieving, i.e., achieving the capacity region of the two-user GMAC-NF. Parallel work of [3] involves extending the classic SK scheme [2] to a broadcast situation; namely, the Gaussian broadcast channel with noiseless feedback (GBC-NF). Ref. [4] proposed an SK-type feedback scheme for the GBC-NF, but unfortunately this scheme is not capacity-achieving. Other applications of SK schemes include the following:

- Weissman and Merhav [5] proposed an SK-type feedback scheme for the dirty paper channel with noiseless feedback and showed that this scheme is capacity-achieving. Subsequently, Rosenzweig [6] extended the SK-type scheme of [5] to multiple-access and broadcast situations.
- Kim extended the SK scheme to colored (non-white) Gaussian channels with noiseless feedback [7,8] and showed that these extended schemes are also capacity-achieving for some special cases.
- Bross [9] extended the SK-type scheme to the Gaussian relay channel with noiseless feedback and showed that the proposed scheme is better than the ones already existing in the literature.
- Ben-Yishai and Shayevitz [10] studied the SK-type scheme for the white Gaussian channel with noisy feedback and showed that a variation of the SK scheme achieves a rate that tends to be the capacity for some special cases.

**Figure 1:**
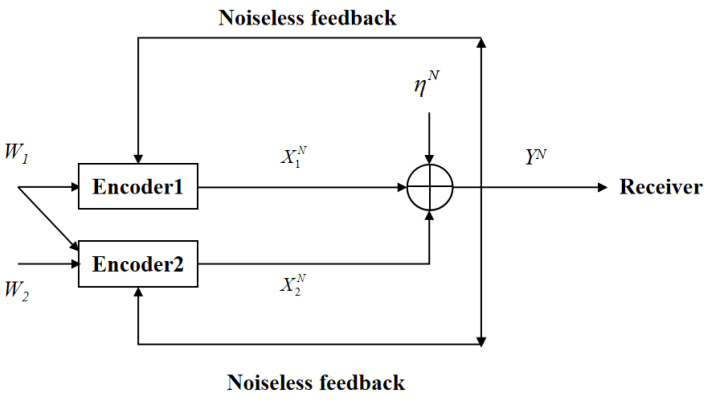
The two-user GMAC with degraded message sets and noiseless feedback.

In this paper, we revisit the two-user GMAC-NF by considering the case that one common message for both users and one private message for an intended user are transmitted through the channel (see Figure 1), which is also called the two-user GMAC with degraded message sets and noiseless feedback (GMAC-DMS-NF). Here, note that the two-user GMAC with degraded message sets is especially useful when considering partial cooperation between the encoders of the GMAC. Although it has already been shown that feedback does not increase the capacity region of the two-user GMAC-DMS-NF, the capacity-achieving SK-type feedback scheme of this model remains open. In this paper, a novel SK-type feedback scheme is proposed for the model of Figure 1 and it is proven to be capacity-achieving.

The rest of this paper is organized as follows. Section 2 introduces some preliminary results for the SK scheme and the GMAC with degraded message sets. Model formulation and the main results are given in Section 3. Conclusions and future work are given in Section 4.

## 2. Preliminary

In this section, we introduce the SK schemes for the point-to-point white Gaussian channel with feedback and the two-user GMAC-NF.

*Basic notation*: A random variable (RV) is denoted with an upper case letter (e.g., *X*), its value is denoted with an lower case letter (e.g., *x*), the finite alphabet of the RV is denoted with calligraphic letter (e.g., X), and the probability distribution of an event {X = x} is denoted with P(x). A random vector and its value are denoted by a similar convention. For example, $X^{N}$ represents an *N*-dimensional random vector $\left(X_{1},\;\ldots ,\;X_{N}\right)$, and $x^{N}\;=\;\left(x_{1},\;\ldots ,\;x_{N}\right)$ represents a vector value in $X^{N}$ (the *N*-th Cartesian power of the finite alphabet X). In addition, define $A_{j}^{N}\;=\;\left(A_{j,1},\;A_{j,2},\;\ldots ,\;A_{j,N}\right)$ and $a_{j}^{N}=\;\left(a_{j,1},\;a_{j,2},\;\ldots ,\;a_{j,N}\right)$. Throughout this paper, the base of the log function is 2.

### 2.1. The SK Scheme for the Point-to-Point White Gaussian Channel with Feedback

**Figure 2:**
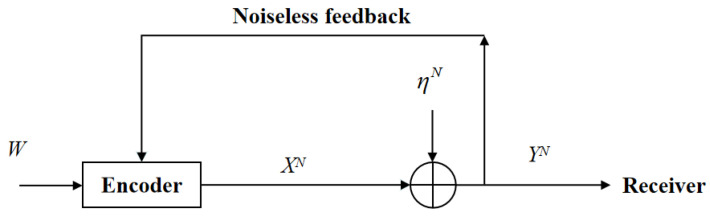
The point-to-point white Gaussian channel with feedback.

For the white Gaussian channel with feedback (see Figure 2), at time instant *i* (i ∈ {1, 2, …, N}), the channel input and output are given by

(1) $$\begin{array}{ccc} & & Y_{i}\;=\;X_{i}\;+\;\eta_{i}, \end{array}$$

where $Y_{i}$ is the channel output, $X_{i}$ is the channel input, and $\eta_{i}\;∼\;N\left(0,\;\sigma^{2}\right)$ is the i.i.d white Gaussian noise across the time index *i*. The message *W* is uniformly chosen from the set W={1, 2, …, |W|}, and the *i*-th time channel input $X_{i}$ is a function of the message *W* and the feedback $Y_{i-1}$, namely,

(2) $$\begin{array}{ccc} & & X_{i}\;=\;f_{i}\left(W,Y^{i-1}\right). \end{array}$$

The receiver obtains an estimation $\hat{W}\;=\;\psi \left(Y^{N}\right)$, where ψ is the receiver’s decoding function, and the average decoding error probability is given by

(3) $$P_{e}\;=\;\frac{1}{\left|W\right|}\sum_{w\in W}Pr\left\{\psi \left(y^{N}\right)\;\neq \;w|w\right\}.$$

For a given positive rate *R*, if for arbitrarily small ϵ and sufficiently large *N*, there exists a channel encoder–decoder such that

(4) $$\begin{array}{ccc} & & \frac{\log|W|}{N}\geq \;R\;-\;\epsilon ,\;\;\;\;P_{e}\;\leq \;\epsilon , \end{array}$$

we say that *R* is achievable. The channel capacity is the maximum over all achievable rates. Denote the capacity of the white Gaussian channel with feedback by $C_{g}^{f}$. Since feedback does not increase $C_{g}^{f}$, it is easy to see that

(5) $$C_{g}^{f}\;=\;C_{g}\;=\;\frac{1}{2}log\left(1\;+\;\frac{P}{\sigma^{2}}\right),$$

where $C_{g}$ is the capacity of the white Gaussian channel (the same model without feedback).

In [2], it has been shown that the classical SK scheme achieves $C_{g}^{f}$, and it consists of the following two properties:

- At time 1, the channel input is a deterministic function of the real transmitted message.
- From time 2 to *N*, the channel input is the linear combination of previous channel noise.

The details of the SK scheme are described below.

Let $W=\{1\;,2\;\ldots ,\;2^{NR}\}$ be the message set of *W*, divide the interval [−0.5, 0.5] into $2^{NR}$ equally spaced sub-intervals, and each sub-interval center corresponds to a value in *W*. The center of the sub-interval with respect to (w.r.t.) *W* is denoted by θ, where the variance of θ approximately equals $\frac{1}{12}$.

At time 1, the transmitter transmits

(6) $$X_{1}\;=\sqrt{12P}\theta .$$

The receiver receives $Y_{1}\;=\;X_{1}\;+\;\eta_{1}$, and obtains an estimation of θ by computing

(7) $${\hat{\theta }}_{1}\;=\frac{Y_{1}}{\sqrt{12P}}=\;\theta \;+\frac{\eta_{1}}{\sqrt{12P}}=\;\theta \;+\;\epsilon_{1},$$

where $\epsilon_{1}\;={\hat{\theta }}_{1}\;-\;\theta \;=\;\frac{\eta_{1}}{\sqrt{12P}}$, and $\alpha_{1}\;≜\;\frac{\sigma^{2}}{12P}$.

At time 2 ≤ k ≤ N, the receiver obtains $Y_{k}\;=\;X_{k}\;+\;\eta_{k}$, and gets an estimation of $\theta_{k}$ by computing

(8) $${\hat{\theta }}_{k}\;={\hat{\theta }}_{k-1}\;-\frac{E[Y_{k}\epsilon_{k-1}]}{E[Y_{k}^{2}]}Y_{k},$$

where

(9) $$\epsilon_{k}\;=\;{\hat{\theta }}_{k}\;-\;\theta ,$$

(9) yields that

(10) $$\epsilon_{k}\;=\;\epsilon_{k-1}\;-\frac{E[Y_{k}\epsilon_{k-1}]}{E[Y_{k}^{2}]}Y_{k}.$$

At time *k* (k ∈ {2, 3, …, N}), the transmitter sends

(11) $$X_{k}\;=\sqrt{\frac{P}{\alpha_{k-1}}}\epsilon_{k-1},$$

where $\alpha_{k-1}\;≜\;Var\left(\epsilon_{k-1}\right)$.

In [2], it has been shown that the decoding error $P_{e}$ of the above coding scheme is upper-bounded by

(12) $$P_{e}\;\leq \;Pr\{|\epsilon_{N}\left|\;>\frac{1}{2|W|-1}\right\}\;\leq \;2Q\left(\frac{1}{2\cdot 2^{NR}}\frac{1}{\sqrt{\alpha_{N}}}\right),$$

where Q(x) is the tail of the unit Gaussian distribution evaluated at *x*, and

(13) $$\alpha_{N}\;=\;\frac{\sigma^{2}}{12P}{\left(\frac{\sigma^{2}}{P+\sigma^{2}}\right)}^{N-1}.$$

From (12) and (13), we conclude that if

(14) $$R\;<\;\frac{1}{2}log\left(1+\frac{P}{\sigma^{2}}\right)=C_{g},$$

$P_{e}\;\to \;0$ as N → ∞.

### 2.2. The Two-User GMAC with Degraded Message Sets

The channel model consisting of two inputs, one output, and the Gaussian channel noise is called GMAC. On the basis of GMAC, if message $W_{1}$ is known by encoder 1 and encoder 2, message 2 is only known by encoder 2, and this model is called GMAC-DMS.

**Figure 3:**
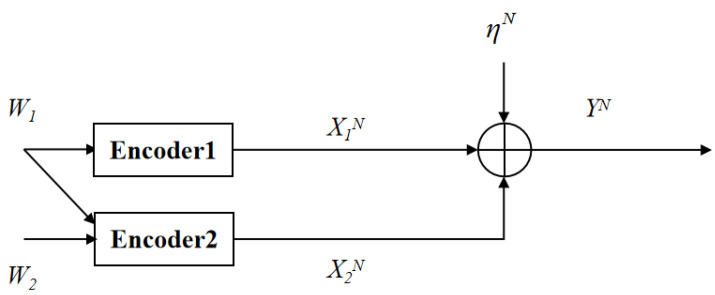
The GMAC with degraded message sets.

The GMAC with degraded message sets is shown in Figure 3. At time *i* (i ∈ {1, 2, …, N}), the channel inputs and output are given by

(15) $$Y_{i}\;=\;X_{1,i}\;+\;X_{2,i}\;+\;\eta_{i},$$

where $X_{1,i}$, $X_{2,i}$ are the channel inputs, respectively, subject to average power constraints $P_{1}$ and $P_{2}$, namely, $\frac{1}{N}\sum_{i=1}^{N}E\left[X_{1,i}^{2}\right]\;\leq \;P_{1}$, $\frac{1}{N}\sum_{i=1}^{N}E\left[X_{2,i}^{2}\right]\;\leq \;P_{2}$, $Y_{i}$ is the channel output, $\eta_{i}\;∼\;N\left(0,\sigma^{2}\right)$ is i.i.d. Gaussian noise across *i*. The message $W_{j}\left(j\;=\;1,\;2\right)$ is uniformly drawn in the set $W_{j}=\left\{1,\;2,\;\ldots ,\;\right|W_{j}\left|\right\}$. The input $X_{1}^{N}$ is a function of the message $W_{1}$, and the input $X_{2}^{N}$ is a function of both messages $W_{1}$ and $W_{2}$. After receiving the channel output, the receiver computes $\left({\hat{W}}_{1},{\hat{W}}_{2}\right)\;=\;\psi \left(Y^{N}\right)$ for decoding, where ψ is the receiver’s decoding function. The average decoding error probability is defined as

(16) $$P_{e}\;=\frac{1}{|W_{1}\left|\cdot \right|W_{2}|}\sum_{w_{1}\in W_{1},w_{2}\in W_{2}}Pr\left\{\psi \left(y^{N}\right)\;\neq \;\left(w_{1},w_{2}\right)|\left(w_{1},w_{2}\right)\right\}.$$

A rate pair $\left(R_{1},R_{2}\right)$ is said to be achievable if, for any ϵ and sufficiently large *N*, there exist channel encoders and decoder such that

(17) $$\frac{log|W_{1}|}{N}\leq \;R_{1}\;-\;\epsilon ,\;\;\;\frac{log|W_{2}|}{N}\leq \;R_{2}\;-\;\epsilon ,\;\;\;\;P_{e}\;\leq \;\epsilon .$$

The capacity region of the GMAC-DMS is noted as $C_{gmac-dms}$, which is composed of all such achievable rate pairs.

**Theorem** **1.** *The capacity region* $C_{gmac-dms}$ *is given by*

(18) $$\begin{array}{ccc} C_{gmac-dms}\;=\underset{0\leq \rho \leq 1}{⋃} & \; & \left\{\left(R_{1}\;\geq \;0,R_{2}\;\geq \;0\right):R_{2}\;\leq \frac{1}{2}\log\left(1\;+\;\frac{P_{2}\left(1-\rho^{2}\right)}{\sigma_{1}^{2}}\right),\right.\\ \; & \; & \left.R_{1}\;+\;R_{2}\;\leq \frac{1}{2}\log\left(1\;+\;\frac{P_{1}+P_{2}+2\sqrt{P_{1}P_{2}}\rho }{\sigma_{1}^{2}}\right)\right\}. \end{array}$$

**Proof.**  Achievability proof of $C_{gmac-dms}$: From [11], the capacity region $C_{mac-dms}$ of the discrete memoryless multiple-access channel with degraded message sets is given by

(19) $$C_{mac-dms}\;=\;\left\{\left(R_{1},\;R_{2}\right)\;:\;R_{2}\;\leq \;I\left(X_{2};Y|X_{1}\right),\;R_{1}\;+\;R_{2}\;\leq \;I\left(X_{1},\;X_{2};Y\right)\right\}$$

for some joint distribution $P(x_{1},\;x_{2})$. Then, substituting $X_{1}\;∼\;N\left(0,\;P_{1}\right)$, $X_{2}\;∼\;N\left(0,\;P_{2}\right)$ and (15) into (19), defining

(20) $$\rho \;=\frac{E[X_{1}X_{2}]}{\sqrt{P_{1}P_{2}}},$$

and following the idea of the encoding–decoding scheme of [11], the achievability of $C_{gmac-dms}$ is proved.

Converse proof of $C_{gmac-dms}$: The converse proof of $C_{gmac-dms}$ follows the converse part of GMAC with feedback [3] (see the converse proof of the bounds on $R_{2}$ and $R_{1}\;+\;R_{2}$), and hence we omit the details here. The proof of Theorem 1 is completed. □

## 3. Model Formulation and the Main Results

In this section, we will first give a formal definition of the two-user GMAC-DMS-NF, then we will give the main results of this paper.

### 3.1. The Two-User GMAC-DMS-NF

The two-user GMAC-DMS-NF is shown in Figure 1. At time *i* (i ∈ {1, 2, …, N}), the channel inputs and output are given by

(21) $$Y_{i}\;=\;X_{1,i}\;+\;X_{2,i}\;+\;\eta_{i},$$

where $X_{1,i}$, $X_{2,i}$ are the channel inputs, respectively, subject to average power constraints $P_{1}$ and $P_{2}$, namely, $\frac{1}{N}\sum_{i=1}^{N}E\left[X_{1,i}^{2}\right]\;\leq \;P_{1}$, $\frac{1}{N}\sum_{i=1}^{N}E\left[X_{2,i}^{2}\right]\;\leq \;P_{2}$, $Y_{i}$ is the channel output, $\eta_{i}\;∼\;N\left(0,\;\sigma^{2}\right)$ is the i.i.d. Gaussian noise across *i*. The message $W_{j}\left(j\;=\;1,\;2\right)$ is uniformly drawn in the set $W_{j}\;=\;\left\{1,\;2,\;\ldots ,\;\right|W_{j}\left|\right\}$. At time *i*, the input $X_{1,i}$ is a function of the common message $W_{1}$ and the feedback $Y^{i-1}$, and the input $X_{2,i}$ is a function of the common message $W_{1}$, the private message $W_{2}$, and the feedback $Y^{i-1}$. After receiving the channel output, the receiver generates an estimation pair $\left({\hat{W}}_{1},{\hat{W}}_{2}\right)\;=\;\psi \left(Y^{N}\right)$, where ψ is the receiver’s decoding function. This model’s average decoding error probability equals

(22) $$P_{e}\;=\frac{1}{|W_{1}\left|\cdot \right|W_{2}|}\sum_{w_{1}\in W_{1},w_{2}\in W_{2}}Pr\left\{\psi \left(y^{N}\right)\;\neq \;\left(w_{1},w_{2}\right)|\left(w_{1},w_{2}\right)\right\}.$$

The capacity region of the two-user GMAC-DMS-NF is noted as $C_{gmac-dms}^{f}$, and it is characterized in the following Theorem 2.

**Theorem** **2.** *$C_{gmac-dms}^{f}\;=\;C_{gmac-dms}$, where $C_{gmac-dms}$ is given in Theorem 1.*

**Proof.**  From the converse proof of the bounds on $R_{2}$ and $R_{1}\;+\;R_{2}$ in [3], we conclude that

(23) $$C_{gmac-dms}^{f}\;\subseteq \;C_{gmac-dms}.$$

However, since the non-feedback model is a special case of the feedback model, thus we have

(24) $$C_{gmac-dms}\;\subseteq \;C_{gmac-dms}^{f}.$$

The proof of Theorem 2 is completed. □

### 3.2. A Capacity-Achieving SK-Type Scheme for the Two-User GMAC-DMS-NF

**Figure 4:**
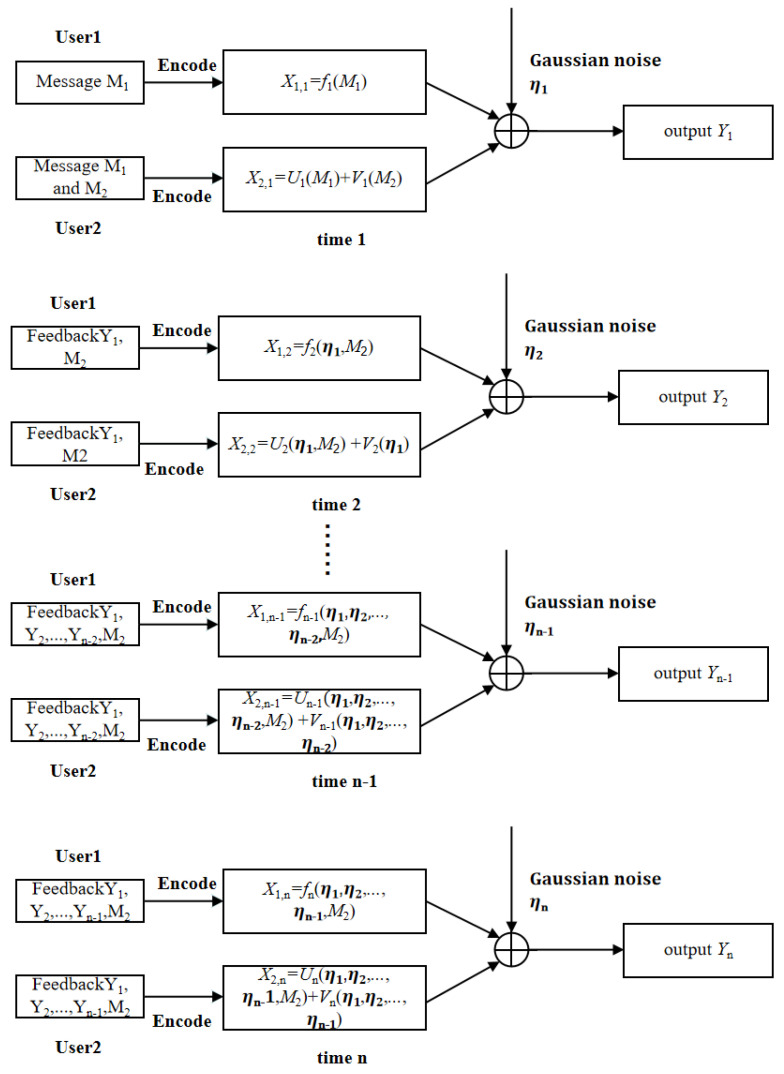
A two-step SK-type scheme for the two-user GMAC-DMS-NF.

In this subsection, we propose a two-step SK-type scheme for the two-user GMAC-DMS-NF, and show that this scheme is capacity-achieving, i.e., achieving $C_{gmac-dms}^{f}$. This new scheme is briefly described in the following Figure 4.

The common message $W_{1}$ is encoded by both transmitters, and the private message $W_{2}$ is only available by Transmitter 2. Transmitter 1 uses power $P_{1}$ to encode $W_{1}$ and the feedback as $X_{1}^{N}$. Transmitter 2 uses power $\left(1\;-\;\rho^{2}\right)P_{2}$ to encode $W_{2}$ and the feedback as $V^{N}$, and power $\rho^{2}P_{2}$ to encode $W_{1}$ and the feedback as $U^{N}$, where 0 ≤ ρ ≤ 1. Here note that since $W_{1}$ is known by Transmitter 2, the codewords $X_{1}^{N}$ and $U^{N}$ can be subtracted when applying the SK scheme to $W_{2}$, i.e., for the SK scheme of $W_{2}$, the equivalent channel model has input $V^{N}$, output $Y^{\prime N}\;=\;Y^{N}\;-\;X_{1}^{N}\;-\;U^{N}$, and channel noise $\eta^{N}$.

In addition, since $W_{1}$ is known by both transmitters and $W_{2}$ is only available at Transmitter 2, for the SK scheme of $W_{1}$, the equivalent channel model has inputs $X_{1}^{N}$ and $U^{N}$, output $Y^{N}$, and channel noise $\eta_{1}^{N}\;+\;V^{N}$, which is non-white Gaussian noise since $V^{N}$ is not i.i.d. generated. Furthermore, we observe that

(25) $$Y_{i}\;=\;X_{1,i}\;+\;U_{i}\;+\;V_{i}\;+\;\eta_{i}\;=\;X_{i}^{*}\;+\;V_{i}\;+\;\eta_{i},$$

where $X_{i}^{*}\;=\;X_{1,i}\;+\;U_{i}$, $X_{i}^{*}$ is Gaussian-distributed with zero mean and variance $P_{i}^{*}$,

(26) $$P_{i}^{*}\;=\;E\left(X_{i}^{*2}\right)\;=\;P_{1}\;+\;\rho^{2}P_{2}\;+\;2\sqrt{P_{1}P_{2}}\rho \rho_{i}^{\prime }\;\leq \;P_{1}\;+\;\rho^{2}P_{2}\;+\;2\sqrt{P_{1}P_{2}}\rho \;=\;P^{*},$$

where

(27) $$\rho_{i}^{\prime }\;=\;\frac{E[X_{1,i}U_{i}]}{\rho \sqrt{P_{1}P_{2}}},$$

and $0\leq \rho_{i}^{\prime }\leq 1$. Hence for the SK scheme of $W_{1}$, the input of the equivalent channel model can be viewed as $X_{i}^{*}$, where $X_{i}^{*}∼N\left(0,P_{i}^{*}\right)$. Define

(28) $$U_{i}\;=\;\rho \sqrt{\frac{P_{2}}{P_{1}}}X_{1,i},$$

Then we have $\rho^{\prime }\;=\;1$, which leads to

(29) $$P_{i}^{*}\;=\;P^{*}\;=\;P_{1}\;+\;\rho^{2}P_{2}\;+\;2\sqrt{P_{1}P_{2}}\rho ,$$

where $X_{i}^{*}∼N\left(0,P^{*}\right)$. The encoding and decoding procedure of Figure 4 is described below.

#### 3.2.1. Encoding Procedure for the Two-Step SK-Type Scheme

Define $W_{j}\;=\;\left\{1,\;2,\;\ldots ,\;2^{NR_{j}}\right\}$ and divide the interval [−0.5, 0.5] into $2^{NR_{j}}$ equally spaced sub-intervals. The center of each sub-interval is mapped to a message value in $W_{j}$ (j=1, 2). Let $\theta_{j}$ be the center of the sub-interval w.r.t. the message $W_{j}$ (the variance of $\theta_{j}$ approximately equals $\frac{1}{12}$).

At time 1, Transmitter 2 sends

(30) $$V_{1}\;=\;\sqrt{12(1-\rho^{2}P_{2})}\theta_{2}.$$

Transmitter 1 and Transmitter 2, respectively, send $X_{1,1}$, $U_{1}\;=\;\rho \sqrt{\frac{P_{2}}{P_{1}}}X_{1,1}$ such that

(31) $$X_{1}^{*}\;=\;U_{1}\;+\;X_{1,1}\;=\;\sqrt{12P^{*}}\theta_{1}.$$

The receiver obtains $Y_{1}\;=\;V_{1}\;+\;X_{1,1}\;+\;U_{1}\;+\;\eta_{1}$ and sends $Y_{1}$ back to Transmitter 2. Subtracting $X_{1,1}$ and $U_{1}$ from $Y_{1}$ and letting $Y_{1}^{\prime }\;=\;V_{1}\;+\;\eta_{1}$, Transmitter 2 computes

(32) $$\frac{Y_{1}^{\prime }}{\sqrt{12(1-\rho^{2}P_{2})}}\;=\;\theta_{2}\;+\;\frac{\eta_{1}}{\sqrt{12(1-\rho^{2}P_{2})}}\;=\;\theta_{2}\;+\;\epsilon_{1},$$

and defines $\alpha_{1}\;≜\;Var\left(\epsilon_{1}\right)\;=\;\frac{\sigma^{2}}{12\left(1-\rho^{2}\right)P_{2}}$. Since $Y_{1}\;=\;X_{1}^{*}\;+\;V_{1}\;+\;\eta_{1}$, Transmitter 1 computes

(33) $$\frac{Y_{1}}{\sqrt{12P^{*}}}\;=\;\frac{U_{1}\;+\;X_{1,1}\;+\;V_{1}\;+\;\eta_{1}}{\sqrt{12P^{*}}}\;=\;\theta_{1}\;+\;\frac{V_{1}\;+\;\eta_{1}}{\sqrt{12P^{*}}}\;=\;\theta_{1}\;+\;\epsilon_{1}^{\prime },$$

and defines

(34) $$\alpha_{1}^{\prime }\;≜\;Var\left(\epsilon_{1}^{\prime }\right)\;=\;\frac{\sigma^{2}\;+\;\left(1-\rho^{2}P_{2}\right)}{12P^{*}}.$$

At time 2, Transmitter 2 sends

(35) $$V_{2}\;=\;\sqrt{\frac{\left(1-\rho^{2}\right)P_{2}}{\alpha_{1}}}\epsilon_{1}.$$

In the meantime, Transmitter 1 and Transmitter 2, respectively, send $X_{1,2}$ and $U_{2}\;=\;\rho \sqrt{\frac{P_{2}}{P_{1}}}X_{1,2}$ such that

(36) $$X_{2}^{*}\;=\;U_{2}\;+\;X_{1,2}\;=\;\sqrt{\frac{P^{*}}{\alpha_{1}^{\prime }}}\epsilon_{1}^{\prime }.$$

At time 3 ≤ k ≤ N, once it has received $Y_{k-1}\;=\;X_{1,k-1}\;+\;U_{k-1}\;+\;V_{k-1}\;+\;\eta_{1,k-1}$, Transmitter 2 computes

(37) $$\epsilon_{k-1}\;=\;\epsilon_{k-2}\;-\;\frac{E[\left(Y_{k-1}\;-\;X_{1,k-1}\;-\;U_{k_{1}}\right)\epsilon_{k-2}]}{E[{\left(Y_{k-1}\;-\;X_{1,k-1}\;-\;U_{k-1}\right)}^{2}]}\left(Y_{k-1}\;-\;X_{1,k-1}\;-\;U_{k-1}\right),$$

and sends

(38) $$V_{k}\;=\;\sqrt{\frac{\left(1\;-\;\rho^{2}\right)P_{2}}{\alpha_{k-1}}}\epsilon_{k-1},$$

where $\alpha_{k-1}\;≜\;Var\left(\epsilon_{k-1}\right)$. In the meantime, Transmitters 1 and 2, respectively, send $X_{1,k}$ and $U_{k}\;=\;\rho \sqrt{\frac{P_{2}}{P_{1}}}X_{1,k}$ such that

(39) $$X_{k}^{*}\;=\;U_{k}\;+\;X_{1,k}\;=\;\sqrt{\frac{P^{*}}{\alpha_{k-1}^{\prime }}}\epsilon_{k-1}^{\prime }.$$

where

(40) $$\epsilon_{k-1}^{\prime }\;=\;\epsilon_{k-2}^{\prime }\;-\;\frac{E[Y_{k-1}\epsilon_{k-2}^{\prime }]}{E[Y_{k-1}^{2}]}Y_{k-1},$$

and $\alpha_{k-1}^{\prime }≜Var\left(\epsilon_{k-1}^{\prime }\right)$.

#### 3.2.2. Decoding Procedure for the Two-Step SK-Type Scheme

The decoding procedure for the receiver consists of two steps. First, from (8), we see that at time (1 ≤ k ≤ N), the receiver’s estimation ${\hat{\theta }}_{1}$ of $W_{1}\left(\theta_{1}\right)$ is given by

(41) $${\hat{\theta }}_{1,k}={\hat{\theta }}_{1,k-1}\;-\;\frac{E[Y_{k}\epsilon_{k-1}^{\prime }]}{E[Y_{k}^{2}]}Y_{k},$$

where $\epsilon_{k-1}^{\prime }\;=\;{\hat{\theta }}_{1,k-1}\;-\;\theta_{1}$ and

(42) $${\hat{\theta }}_{1,1}\;=\;\frac{Y_{1}}{\sqrt{12P^{*}}}\;=\;\frac{U_{1}\;+\;X_{1,1}\;+\;V_{1}\;+\;\eta_{1}}{\sqrt{12P^{*}}}\;=\;\theta_{1}\;+\;\frac{V_{1}\;+\;\eta_{1}}{\sqrt{12P^{*}}}\;=\;\theta_{1}\;+\;\epsilon_{1}^{\prime }.$$

Second, after decoding $W_{1}$ and the corresponding codewords $X_{1,k}$ and $U_{k}$ for all 1 ≤ k ≤ N, the receiver subtracts $X_{1,k}$ and $U_{k}$ from $Y_{k}$, and obtains $Y_{k}^{\prime }\;=\;V_{k}\;+\;\eta_{1,k}$. At time 1 ≤ k ≤ N, the receiver computes ${\hat{\theta }}_{2,k}$ of $W_{2}\left(\theta_{2}\right)$ by

(43) $${\hat{\theta }}_{2,k}\;=\;{\hat{\theta }}_{2,k-1}\;-\;\frac{E[Y_{k}^{\prime }\epsilon_{k-1}]}{E[{\left(Y_{k}^{\prime }\right)}^{2}]}Y_{k}^{\prime },$$

where $\epsilon_{k-1}\;=\;{\hat{\theta }}_{2,k-1}\;-\;\theta_{2}$ and

(44) $${\hat{\theta }}_{2,1}\;=\;\frac{Y_{1}^{\prime }}{\sqrt{12\left(1-\rho^{2}\right)P_{2}}}\;=\;\theta_{2}\;+\;\frac{\eta_{1}}{\sqrt{12\left(1-\rho^{2}\right)P_{2}}}\;=\;\theta_{2}\;+\;\epsilon_{1}.$$

The receiver’s decoding error probability $P_{ej}\left(j\;=\;1,\;2\right)$ for $W_{j}$ is upper-bounded by

(45) $$P_{e}\;\leq \;P_{e1}\;+\;P_{e2}.$$

From the classical SK scheme [2] (also introduced in Section 2.1), we know that the decoding error probability $P_{e2}$ of $W_{2}$ tends to 0 as N → ∞ if

(46) $$R_{2}\;\leq \;\frac{1}{2}log\left(1\;+\;\frac{\left(1-\rho_{2}\right)P_{2}}{\sigma^{2}}\right),$$

and hence we omit the derivation here. The decoding error probability $P_{e1}$ is upper-bounded by the following Lemma 1.

**Lemma** **1.** $P_{e1}\;\to \;0$ as N → ∞ if $R_{1}\;\leq \;\frac{1}{2}log\left(1\;+\;\frac{P^{*}}{\left(1-\rho^{2}\right)P_{2}+\sigma^{2}}\right)$ is satisfied.

**Proof.**  See Appendix A. □

From (46) and Lemma 1, we can conclude that if $R_{1}\;\leq \;\frac{1}{2}\log\left(1+\frac{P_{1}+\rho^{2}P_{2}+2\sqrt{P_{1}P_{2}}}{\left(1-\rho^{2}\right)P_{2}+\sigma^{2}}\right),R_{2}\leq \frac{1}{2}\log\left(1+\frac{\left(1-\rho^{2}\right)P_{2}}{\sigma^{2}}\right)$, the decoding error probability $P_{e}$ of the receiver tends to 0 as N → ∞. In other words, the rate pair $\left(R_{1}\;=\;\frac{1}{2}\log\left(1+\frac{P_{1}+\rho^{2}P_{2}+2\sqrt{P_{1}P_{2}}}{\left(1-\rho^{2}\right)P_{2}+\sigma^{2}}\right),R_{2}=\frac{1}{2}\log\left(1+\frac{\left(1-\rho^{2}\right)P_{2}}{\sigma^{2}}\right)\right)$ is achievable for all 0 ≤ ρ ≤ 1, which indicates that all rate pairs $\left(R_{1},\;R_{2}\right)$ in $C_{gmac-dms}^{f}$ are achievable. Hence this two-step SK-type feedback scheme achieves the capacity region $C_{gmac-dms}^{f}$ of the two-user GMAC-DMS-NF.

## 4. Conclusions

In this paper, we have proposed a capacity-achieving SK-type feedback scheme for the two-user GMAC-DMS-NF, which remains open in the literature. The proposed scheme in this paper adopts a two-step encoding–decoding procedure, where the common message is encoded as the codeword $X_{1}^{N}$ and one part of the codeword $X_{2}^{N}$ (namely, $U^{N}$), the private message is encoded as the other part of the codeword $X_{2}^{N}$ (namely, $V^{N}$), and the SK scheme is applied to the encoding procedure of both common and private messages. In the decoding procedure, the receiver first decodes the common message by using the SK decoding scheme and viewing $V^{N}$ as part of the channel noise. Then, after successfully decoding the common message, the receiver subtracts its corresponding codewords $X_{1}^{N}$ and $U^{N}$ from its received signal $Y^{N}$, and uses the SK decoding scheme to decode the private message. Here note that the proposed two-step SK-type scheme is not a trivial extension of the already existing feedback scheme [3] for the two-user GMAC, where two independent encoders apply the SK scheme to encode their independent messages. In fact, a simple application of Ozarow’s SK-type scheme [3] cannot achieve the capacity region of the two-user GMAC-DMS-NF, which indicates that it is not an optimal choice for the two-user GMAC-DMS-NF. Possible future work includes the following:

- Capacity-achieving SK-type feedback schemes for the fading GMAC.
- SK-type feedback schemes for the GMAC with noisy feedback.

## Data Availability

The data used in this work are available from the corresponding author upon reasonable request.

## Abbreviations

The following abbreviations are used in this manuscript:

SK	Schalkwijk–Kailath
GMAC	Gaussian multiple-access channel
GMAC-NF	Gaussian multiple-access channel with noiseless feedback
DMC	Discrete memoryless channel
GBC-NF	Gaussian broadcast channel with noiseless feedback
GMAC-DMS	Two-user GMAC with degraded message sets
GMAC-DMS-NF	Two-user GMAC with degraded message sets and noiseless feedback

## Appendix A

Appendix A proves Lemma 1 described in Section 3. First, we determine the channel noise of the equivalent channel model. For the SK scheme of $W_{1}$, the equivalent channel model has input $X^{*N}\;=\;X_{1}^{N}\;+\;U^{N}$, output $Y^{N}$, and channel noise $\eta_{1}^{N}\;+\;V^{N}$, where $\eta^{N}\;+\;V^{N}$ is non-white Gaussian because $V^{N}$ is generated by the classical SK scheme and it is a combination of previous channel noise. For 1 ≤ k ≤ N, define

(A1) $$\eta_{k}^{\prime }\;=\;\eta_{k}\;+\;V_{k}.$$

Note that

(A2) $$\begin{array}{ccc} \; & \; & E\left[{\left(\eta_{k}^{\prime }\right)}^{2}\right)]\;=\;E\left[{\left(\eta_{k}\;+\;V_{k}\right)}^{2}\right]\\ \; & \; & \overset{\left(a\right)}{=}E\left[{\left(\eta_{k}\right)}^{2}\right]\;+\;E\left[{\left(V_{k}\right)}^{2}\right]\overset{\left(b\right)}{=}\sigma^{2}\;+\;\left(1-\rho^{2}\right)P_{2}, \end{array}$$

where (a) follows from the fact that $V_{k}$ is independent of $\eta_{k}$ since $V_{1}$ is a function of $\theta_{2}$ and $V_{k}\left(2\leq k\leq N\right)$ is a function of $\eta_{1},\;\ldots ,\;\eta_{k-1}$, and (b) follows from (38). Furthermore, from (37) and (38), $V_{k}$ can be re-written as

(A3) $$\begin{array}{ccc} \; & \; & V_{k}\;=\;\sqrt{\frac{\left(1-\rho^{2}\right)P_{2}}{\alpha_{k-1}}}\epsilon_{k-1}\\ \; & \; & =\sqrt{\frac{\left(1-\rho^{2}\right)P_{2}}{\alpha_{k-1}}}\left(\epsilon_{k-2}\;-\;\frac{E[\left(V_{k-1}+\eta_{k-1}\right)\epsilon_{k-2}]}{E[\left(V_{k-1}\;+\;\eta_{k-1}^{2}\right)]}\left(V_{k-1}+\eta_{k-1}\right)\right)\\ \; & \; & \overset{\left(c\right)}{=}\sqrt{\frac{\left(1-\rho^{2}\right)P_{2}}{\alpha_{k-1}}}\left(\epsilon_{k-2}\;-\;\frac{\sqrt{\left(1-\rho^{2}\right)P_{2}\alpha_{k-2}}}{\left(1-\rho^{2}\right)P_{2}+\sigma_{1}^{2}}\left(V_{k-1}\;+\;\eta_{k-1}\right)\right)\\ \; & \; & =\sqrt{\frac{\left(1-\rho^{2}\right)P_{2}}{\alpha_{k-2}}}\sqrt{\frac{\alpha_{k-2}}{\alpha_{k-1}}}\left(\epsilon_{k-2}\;-\;\frac{\sqrt{\left(1-\rho^{2}\right)P_{2}\alpha_{k-2}}}{\left(1-\rho^{2}\right)P_{2}+\sigma_{1}^{2}}\left(V_{k-1}\;+\;\eta_{k-1}\right)\right)\\ \; & \; & \overset{\left(d\right)}{=}\sqrt{\frac{\alpha_{k-2}}{\alpha_{k-1}}}V_{k-1}\;-\;\sqrt{\frac{\left(1-\rho^{2}\right)P_{2}}{\alpha_{k-1}}}\frac{\sqrt{\left(1-\rho^{2}\right)P_{2}\alpha_{k-2}}}{\left(1-\rho^{2}\right)P_{2}+\sigma_{1}^{2}}\left(V_{k-1}\;+\;\eta_{k-1}\right)\\ \; & \; & =\sqrt{\frac{\alpha_{k-2}}{\alpha_{k-1}}}\frac{\sigma_{1}^{2}}{\left(1-\rho^{2}\right)P_{2}+\sigma_{1}^{2}}V_{k-1}\;-\;\sqrt{\frac{\alpha_{k-2}}{\alpha_{k-1}}}\frac{\left(1-\rho^{2}\right)P_{2}}{\left(1-\rho^{2}\right)P_{2}+\sigma_{1}^{2}}\eta_{k-1}. \end{array}$$

where (c) follows from $\epsilon_{k-2}$ and is independent of $\eta_{k-1}$, $V_{k-1}\;=\;\sqrt{\frac{\left(1-\rho^{2}\right)P_{2}}{\alpha_{k-2}}}\epsilon_{k-2}$, $\alpha_{k-2}\;≜\;Var\left(\epsilon_{k-2}\right)$, and (d) follows from $V_{k-1}\;=\;\sqrt{\frac{\left(1-\rho^{2}\right)P_{2}}{\alpha_{k-2}}}\epsilon_{k-2}$. Substituting (A3) into (A1), we have

(A4) $$\begin{array}{ccc} \; & \; & \eta_{k}^{\prime }\;=\;\eta_{k}\;+\;V_{k}\;=\;\eta_{k}\;+\;\sqrt{\frac{\alpha_{k-2}}{\alpha_{k-1}}}\frac{\sigma^{2}}{\left(1-\rho^{2}\right)P_{2}+\sigma^{2}}V_{k-1}\;-\;\sqrt{\frac{\alpha_{k-2}}{\alpha_{k-1}}}\frac{\left(1-\rho^{2}\right)P_{2}}{\left(1-\rho^{2}\right)P_{2}+\sigma^{2}}\eta_{k-1}\\ \; & \; & =\;\eta_{k}\;+\;\sqrt{\frac{\alpha_{k-2}}{\alpha_{k-1}}}\frac{\sigma^{2}}{\left(1-\rho^{2}\right)P_{2}+\sigma^{2}}V_{k-1}\;-\;\sqrt{\frac{\alpha_{k-2}}{\alpha_{k-1}}}\frac{\left(1-\rho^{2}\right)P_{2}}{\left(1-\rho^{2}\right)P_{2}+\sigma^{2}}\eta_{k-1}\\ \; & \; & +\;\sqrt{\frac{\alpha_{k-2}}{\alpha_{k-1}}}\frac{\sigma^{2}}{\left(1-\rho^{2}\right)P_{2}+\sigma^{2}}\eta_{k-1}\;-\;\sqrt{\frac{\alpha_{k-2}}{\alpha_{k-1}}}\frac{\sigma^{2}}{\left(1-\rho^{2}\right)P_{2}+\sigma^{2}}\eta_{k-1}\\ \; & \; & =\;\eta_{k}\;+\;\sqrt{\frac{\alpha_{k-2}}{\alpha_{k-1}}}\frac{\sigma^{2}}{\left(1-\rho^{2}\right)P_{2}+\sigma^{2}}\left(V_{k-1}\;+\;\eta_{k-1}\right)\;-\;\sqrt{\frac{\alpha_{k-2}}{\alpha_{k-1}}}\eta_{k-1}\\ \; & \; & =\;\eta_{k}\;+\;\sqrt{\frac{\alpha_{k-2}}{\alpha_{k-1}}}\frac{\sigma^{2}}{\left(1-\rho^{2}\right)P_{2}+\sigma^{2}}\eta_{k-1}^{\prime }\;-\;\sqrt{\frac{\alpha_{k-2}}{\alpha_{k-1}}}\eta_{k-1}. \end{array}$$

From the classical SK scheme [2] (see (13)), we know that

(A5) $$\frac{\alpha_{k}}{\alpha_{k-1}}\;=\;\frac{\sigma^{2}}{\left(1\;-\;\rho^{2}\right)P_{2}+\sigma^{2}}.$$

Substituting (A5) into (A4), we obtain

(A6) $$\eta_{k}^{\prime }\;=\;\frac{\sigma }{\sqrt{\left(1-\rho^{2}\right)P_{2}+\sigma^{2}}}\eta_{k-1}^{\prime }\;+\;\eta_{k}\;-\;\sqrt{\frac{\left(1-\rho^{2}\right)P_{2}+\sigma^{2}}{\sigma^{2}}}\eta_{k-1}.$$

Here note that (A6) holds for 3 ≤ k ≤ N, and

(A7) $$\eta_{1}^{\prime }\;=\;\eta_{1}\;+\;\sqrt{12\left(1-\rho^{2}\right)P_{2}}\theta_{2},\;\;\;\eta_{2}^{\prime }\;=\;\eta_{2}\;+\;\frac{\eta_{1}\sqrt{\left(1-\rho^{2}\right)P_{2}}}{\sigma }.$$

After determining the noise expression $\eta_{k}^{\prime }$ of the equivalent channel model, we still need to determine the decoding error $\epsilon_{k}^{\prime }$. According to (40), we have

(A8) $$\begin{array}{ccc} \; & \; & E\left[Y_{k-1}\epsilon_{k-2}^{\prime }\right]=E\left[\left(X_{k-1}^{*}+\eta_{k-1}^{\prime }\right)\epsilon_{k-2}^{\prime }\right]\\ \; & \; & \overset{\left(e\right)}{=}E\left[\left(\sqrt{\frac{P^{*}}{\alpha_{k-2}^{\prime }}}\epsilon_{k-2}^{\prime }+\eta_{k-1}^{\prime }\right)\epsilon_{k-2}^{\prime }\right]\\ \; & \; & =\sqrt{P^{*}\alpha_{k-2}^{\prime }}+E\left[\eta_{k-1}^{\prime }\epsilon_{k-2}^{\prime }\right], \end{array}$$

and

(A9) $$\begin{array}{ccc} \; & \; & E\left[Y_{k-1}^{2}\right]=E\left[{\left(X_{k-1}^{*}+\eta_{k-1}^{\prime }\right)}^{2}\right]=E\left[{\left(\sqrt{\frac{P^{*}}{\alpha_{k-2}^{\prime }}}\epsilon_{k-2}^{\prime }+\eta_{k-1}^{\prime }\right)}^{2}\right]\\ \; & \; & \overset{\left(f\right)}{=}P^{*}+2\sqrt{\frac{P^{*}}{\alpha_{k-2}^{\prime }}}E\left[\epsilon_{k-2}^{\prime }\eta_{k-1}^{\prime }\right]+\left(1-\rho^{2}\right)P_{2}+\sigma^{2}, \end{array}$$

where (e) follows from (39), and (f) follows from (A2). Substituting (A8) and (A9) into (40), $\epsilon_{k-1}^{\prime }$ can be re-written as

$$\begin{array}{ccc} \; & \; & \epsilon_{k-1}^{\prime }=\epsilon_{k-2}^{\prime }-\frac{E[Y_{k-1}\epsilon_{k-2}^{\prime }]}{E[Y_{k-1}^{2}]}Y_{k-1}\\ \; & \; & =\epsilon_{k-2}^{\prime }-\frac{\sqrt{P^{*}\alpha_{k-2}^{\prime }}+E\left[\eta_{k-1}^{\prime }\epsilon_{k-2}^{\prime }\right]}{P^{*}+2\sqrt{\frac{P^{*}}{\alpha_{k-2}^{\prime }}}E\left[\epsilon_{k-2}^{\prime }\eta_{k-1}^{\prime }\right]+\left(1-\rho^{2}\right)P_{2}+\sigma^{2}}\left(\sqrt{\frac{P^{*}}{\alpha_{k-2}^{\prime }}}\epsilon_{k-2}^{\prime }+\eta_{k-1}^{\prime }\right)\\ \; & \; & =\epsilon_{k-2}^{\prime }-\frac{\epsilon_{k-2}^{\prime }\left(P^{*}+E\left[\epsilon_{k-2}^{\prime }\eta_{k-1}^{\prime }\right]\sqrt{\frac{P^{*}}{\alpha_{k-2}^{\prime }}}\right)+\eta_{k-1}^{\prime }\left(\sqrt{P^{*}\alpha_{k-2}^{\prime }+}E\left[\epsilon_{k-2}^{\prime }\eta_{k-1}^{\prime }\right]\right)}{P^{*}+2\sqrt{\frac{P^{*}}{\alpha_{k-2}^{\prime }}}E\left[\epsilon_{k-2}^{\prime }\eta_{k-1}^{\prime }\right]+\left(1-\rho^{2}\right)P_{2}+\sigma^{2}}\\ \; & \; & =\epsilon_{k-2}^{\prime }\frac{\sqrt{\frac{P^{*}}{\alpha_{k-2}^{\prime }}}E\left[\epsilon_{k-2}^{\prime }\eta_{k-1}^{\prime }\right]+\left(1-\rho^{2}\right)P_{2}+\sigma^{2}}{P^{*}+2\sqrt{\frac{P^{*}}{\alpha_{k-2}^{\prime }}}E\left[\epsilon_{k-2}^{\prime }\eta_{k-1}^{\prime }\right]+\left(1-\rho^{2}\right)P_{2}+\sigma^{2}}\\ \; & \; & -\eta_{k-1}^{\prime }\frac{\sqrt{P^{*}\alpha_{k-2}^{\prime }}+E\left[\epsilon_{k-2}^{\prime }\eta_{k-1}^{\prime }\right]}{P^{*}+2\sqrt{\frac{P^{*}}{\alpha_{k-2}^{\prime }}}E\left[\epsilon_{k-2}^{\prime }\eta_{k-1}^{\prime }\right]+\left(1-\rho^{2}\right)P_{2}+\sigma^{2}}. \end{array}$$

From (A10), we see that $\epsilon_{k-1}^{\prime }$ depends on $E[\epsilon_{k-2}^{\prime }\eta_{k-1}^{\prime }]$. Combining (A6) with (A10), we can conclude that

(A10) $$\begin{array}{ccc} \; & \; & E\left[\epsilon_{k-2}^{\prime }\eta_{k-1}^{\prime }\right]=E[\left(\frac{\sigma }{\sqrt{\left(1-\rho^{2}\right)P_{2}+\sigma^{2}}}\eta_{k-1}^{\prime }+\eta_{k}^{\prime }-\sqrt{\frac{\left(1-\rho^{2}\right)P_{2}+\sigma^{2}}{\sigma^{2}}}\eta_{k-1}\right)\\ \; & \; & \cdot (\epsilon_{k-2}^{\prime }\frac{\sqrt{\frac{P^{*}}{\alpha_{k-2}^{\prime }}}E\left[\epsilon_{k-2}^{\prime }\eta_{k-1}^{\prime }\right]+\left(1-\rho^{2}\right)P_{2}+\sigma^{2}}{P^{*}+2\sqrt{\frac{P^{*}}{\alpha_{k-2}^{\prime }}}E\left[\epsilon_{k-2}^{\prime }\eta_{k-1}^{\prime }\right]+\left(1-\rho^{2}\right)P_{2}+\sigma^{2}}\\ \; & \; & -\eta_{k-1}^{\prime }\frac{\sqrt{P^{*}\alpha_{k-2}^{\prime }}+E\left[\epsilon_{k-2}^{\prime }\eta_{k-1}^{\prime }\right]}{P^{*}+2\sqrt{\frac{P^{*}}{\alpha_{k-2}^{\prime }}}E\left[\epsilon_{k-2}^{\prime }\eta_{1,k-1}^{\prime }\right]+\left(1-\rho^{2}\right)P_{2}+\sigma^{2}})]\\ \; & \; & \overset{\left(g\right)}{=}\frac{\sqrt{\frac{P^{*}}{\alpha_{k-2}^{\prime }}}E\left[\epsilon_{k-2}^{\prime }\eta_{k-1}^{\prime }\right]+\left(1-\rho^{2}\right)P_{2}+\sigma^{2}}{P^{*}+2\sqrt{\frac{P^{*}}{\alpha_{k-2}^{\prime }}}E\left[\epsilon_{k-2}^{\prime }\eta_{k-1}^{\prime }\right]+\left(1-\rho^{2}\right)P_{2}+\sigma^{2}}\cdot \frac{\sigma_{1}}{\sqrt{\left(1-\rho^{2}\right)P_{2}+\sigma^{2}}}E\left[\epsilon_{k-2}^{\prime }\eta_{k-1}^{\prime }\right]\\ \; & \; & -\frac{\sqrt{P^{*}\alpha_{k-2}^{\prime }}+E\left[\epsilon_{k-2}^{\prime }\eta_{k-1}^{\prime }\right]}{P^{*}+2\sqrt{\frac{P^{*}}{\alpha_{k-2}^{\prime }}}E\left[\epsilon_{k-2}^{\prime }\eta_{k-1}^{\prime }\right]+\left(1-\rho^{2}\right)P_{2}+\sigma^{2}}\cdot \frac{\sigma }{\sqrt{\left(1-\rho^{2}\right)P_{2}+\sigma^{2}}}E\left[{\left(\eta_{k-1}^{\prime }\right)}^{2}\right]\\ \; & \; & +\frac{\sqrt{P^{*}\alpha_{k-2}^{\prime }}+E\left[\epsilon_{k-2}^{\prime }\eta_{k-1}^{\prime }\right]}{P^{*}+2\sqrt{\frac{P^{*}}{\alpha_{k-2}^{\prime }}}E\left[\epsilon_{k-2}^{\prime }\eta_{k-1}^{\prime }\right]+\left(1-\rho^{2}\right)P_{2}+\sigma^{2}}\cdot \sqrt{\frac{\left(1-\rho^{2}\right)P_{2}+\sigma^{2}}{\sigma^{2}}}E\left[\eta_{k-1}\eta_{k-1}^{\prime }\right]\\ \; & \; & \overset{\left(h\right)}{=}\frac{\sqrt{\frac{P^{*}}{\alpha_{k-2}^{\prime }}}E\left[\epsilon_{k-2}^{\prime }\eta_{k-1}^{\prime }\right]+\left(1-\rho^{2}\right)P_{2}+\sigma^{2}}{P^{*}+2\sqrt{\frac{P^{*}}{\alpha_{k-2}^{\prime }}}E\left[\epsilon_{k-2}^{\prime }\eta_{k-1}^{\prime }\right]+\left(1-\rho^{2}\right)P_{2}+\sigma^{2}}\cdot \frac{\sigma }{\sqrt{\left(1-\rho^{2}\right)P_{2}+\sigma^{2}}}E\left[\epsilon_{k-2}^{\prime }\eta_{k-1}^{\prime }\right]\\ \; & \; & -\frac{\sqrt{P^{*}\alpha_{k-2}^{\prime }}+E\left[\epsilon_{k-2}^{\prime }\eta_{k-1}^{\prime }\right]}{P^{*}+2\sqrt{\frac{P^{*}}{\alpha_{k-2}^{\prime }}}E\left[\epsilon_{k-2}^{\prime }\eta_{k-1}^{\prime }\right]+\left(1-\rho^{2}\right)P_{2}+\sigma^{2}}\cdot \sigma \sqrt{\left(1-\rho^{2}\right)P_{2}+\sigma^{2}}\\ \; & \; & +\frac{\sqrt{P^{*}\alpha_{k-2}^{\prime }}+E\left[\epsilon_{k-2}^{\prime }\eta_{k-1}^{\prime }\right]}{P^{*}+2\sqrt{\frac{P^{*}}{\alpha_{k-2}^{\prime }}}E\left[\epsilon_{k-2}^{\prime }\eta_{k-1}^{\prime }\right]+\left(1-\rho^{2}\right)P_{2}+\sigma^{2}}\cdot \sigma \sqrt{\left(1-\rho^{2}\right)P_{2}+\sigma^{2}}\\ \; & \; & =\frac{\sqrt{\frac{P^{*}}{\alpha_{k-2}^{\prime }}}E\left[\epsilon_{k-2}^{\prime }\eta_{k-1}^{\prime }\right]+\left(1-\rho^{2}\right)P_{2}+\sigma^{2}}{P^{*}+2\sqrt{\frac{P^{*}}{\alpha_{k-2}^{\prime }}}E\left[\epsilon_{k-2}^{\prime }\eta_{k-1}^{\prime }\right]+\left(1-\rho^{2}\right)P_{2}+\sigma^{2}}\cdot \frac{\sigma }{\sqrt{\left(1-\rho^{2}\right)P_{2}+\sigma^{2}}}E\left[\epsilon_{k-2}^{\prime }\eta_{k-1}^{\prime }\right], \end{array}$$

where (g) follows from $E\left[\epsilon_{k-2}^{\prime }\eta_{k-1}\right]=E\left[\epsilon_{k-2}^{\prime }\eta_{k-1}\right]=E\left[\eta_{k-1}^{\prime }\eta_{k}\right]=0$, and (h) follows from (A6), which indicates that

(A11) $$\begin{array}{ccc} \; & \; & E\left[\eta_{k-1}^{\prime }\eta_{k-1}\right]=E\left[\left(\frac{\sigma }{\sqrt{\left(1-\rho^{2}\right)P_{2}+\sigma^{2}}}\eta_{k-2}^{\prime }+\eta_{k-1}-\sqrt{\frac{\left(1-\rho^{2}\right)P_{2}+\sigma^{2}}{\sigma^{2}}}\eta_{k-2}\right)\eta_{k-1}\right]\\ \; & \; & \overset{\left(i\right)}{=}E\left[{\left(\eta_{k-1}\right)}^{2}\right]=\sigma^{2}, \end{array}$$

where (i) follows from $E\left[\eta_{k-2}^{\prime }\eta_{k-1}\right]=E\left[\eta_{k-2}\eta_{k-1}\right]=0$. From (A10) and the fact that

(A12) $$E\left[\epsilon_{1}^{\prime }\eta_{2}^{\prime }\right]=\sqrt{\frac{\left(1-\rho^{2}\right)P_{2}\sigma^{2}}{12P^{*}}}\geq 0,$$

it is easy to see that

(A13) $$E[\epsilon_{k-1}^{\prime }\eta_{k}^{\prime }]\geq 0$$

for all 2≤k≤N. The final step before we bound $P_{e1}$ is the determination of $\alpha_{k}^{\prime }$, which is defined as $\alpha_{k}^{\prime }=Var\left(\epsilon_{k}^{\prime }\right)=E\left[{\left(\epsilon_{k}^{\prime }\right)}^{2}\right]$. Using (A10), we have

(A14) $$\begin{array}{ccc} \; & \; & \alpha_{k}^{\prime }\overset{\left(j\right)}{=}\alpha_{k-1}^{\prime }{\left(\frac{\sqrt{\frac{P^{*}}{\alpha_{k-1}^{\prime }}}E\left[\epsilon_{k-1}^{\prime }\eta_{k}^{\prime }\right]+\left(1-\rho^{2}\right)P_{2}+\sigma^{2}}{P^{*}+2\sqrt{\frac{P^{\prime }}{\alpha_{k-1}^{\prime }}}E\left[\epsilon_{k-1}^{\prime }\eta_{k}^{\prime }\right]+\left(1-\rho^{2}\right)P_{2}+\sigma^{2}}\right)}^{2}\\ \; & \; & -2\frac{\left(\sqrt{\frac{P^{*}}{\alpha_{k-1}^{\prime }}}E\left[\epsilon_{k-1}^{\prime }\eta_{k}^{\prime }\right]+\left(1-\rho^{2}\right)P_{2}+\sigma^{2}\right)\left(\sqrt{P^{*}\cdot \alpha_{k-1}^{\prime }}+E\left[\epsilon_{k-1}^{\prime }\eta_{k}^{\prime }\right]\right)}{{\left(P^{*}+2\sqrt{\frac{P^{\prime }}{\alpha_{k-1}^{\prime }}}E\left[\epsilon_{k-1}^{\prime }\eta_{k}^{\prime }\right]+\left(1-\rho^{2}\right)P_{2}+\sigma^{2}\right)}^{2}}\\ \; & \; & +\left(\sigma^{2}+\left(1-\rho^{2}\right)P_{2}\right){\left(\frac{\sqrt{P^{*}\cdot \alpha_{k-1}^{\prime }}+E\left[\epsilon_{k-1}^{\prime }\eta_{k}^{\prime }\right]}{P^{*}+2\sqrt{\frac{P^{*}}{\alpha_{k-1}^{\prime }}E\left[\epsilon_{k-1}^{\prime }\eta_{k}^{\prime }\right]+\left(1-\rho^{2}\right)P_{2}+\sigma^{2}}}\right)}^{2}\\ \; & \; & \overset{\left(k\right)}{=}\frac{\alpha_{k-1}^{\prime }r^{2}\left(r^{2}+P^{*}\right)+2\sqrt{P^{*}\alpha_{k-1}^{\prime }}r^{2}A_{k}-A_{k}^{2}\left(P^{*}+r^{2}\right)-2\sqrt{\frac{P^{*}}{\alpha_{k-1}^{\prime }}}A_{k}^{3}}{{\left(P^{*}+r^{2}+2\sqrt{\frac{P^{*}}{\alpha_{k-1}^{\prime }}}A_{k}\right)}^{2}}\\ \; & \; & \overset{\left(l\right)}{\leq }\frac{\alpha_{k-1}^{\prime }r^{2}\left(r^{2}+P^{*}\right)+2\sqrt{P^{*}\alpha_{k-1}^{\prime }}r^{2}A_{k}}{{\left(P^{*}+r^{2}+2\sqrt{\frac{P^{*}}{\alpha_{k-1}^{\prime }}}A_{k}\right)}^{2}}\\ \; & \; & =r^{2}\cdot \frac{2\sqrt{P^{*}\alpha_{k-1}^{\prime }}A_{k}+\alpha_{k-1}^{\prime }\left(r^{2}+P^{*}\right)}{{\left(P^{*}+r^{2}+2\sqrt{\frac{P^{*}}{\alpha_{k-1}^{\prime }}}A_{k}\right)}^{2}}\\ \; & \; & =r^{2}\cdot \frac{{\left(\sqrt{r^{2}+P^{*}}\sqrt{\alpha_{k-1}^{\prime }}+\frac{\sqrt{P^{*}}A_{k}}{\sqrt{r^{2}+P^{*}}}\right)}^{2}-\frac{P^{*}A_{k}^{2}}{r^{2}+P^{*}}}{{\left(P^{*}+r^{2}+2\sqrt{\frac{P^{*}}{\alpha_{k-1}^{\prime }}}A_{k}\right)}^{2}}\\ \; & \; & \leq r^{2}\cdot \frac{{\left(\sqrt{r^{2}+P^{*}}\sqrt{\alpha_{k-1}^{\prime }}+\frac{\sqrt{P^{*}}A_{k}}{\sqrt{r^{2}+P^{*}}}\right)}^{2}}{{\left(P^{*}+r^{2}+2\sqrt{\frac{P^{*}}{\alpha_{k-1}^{\prime }}}A_{k}\right)}^{2}}, \end{array}$$

where (j) follows from (A2), (k) follows from the definitions

(A15) $$r\;=\;\sqrt{\left(1-\rho^{2}\right)P_{2}+\sigma^{2}},\;A_{k}\;=\;E\left[\epsilon_{k-1}^{\prime }\eta_{k}^{\prime }\right],$$

and (l) follows from (A13) that $A_{k}\;=\;E\left[\epsilon_{k-1}^{\prime }\eta_{k}^{\prime }\right]\geq 0$. Since $A_{k}\geq 0$ and r≥0, (A14) can be re-written as

(A16) $$\begin{array}{ccc} \; & \; & \sqrt{\alpha_{k}^{\prime }}\leq r\cdot \frac{\sqrt{r^{2}+P^{*}}\sqrt{\alpha_{k-1}^{\prime }}+\frac{\sqrt{P^{*}}A_{k}}{\sqrt{r^{2}+P^{*}}}}{P^{*}+r^{2}+2\sqrt{\frac{P^{*}}{\alpha_{k-1}^{\prime }}}A_{k}}\\ \; & \; & =r\sqrt{\alpha_{k-1}^{\prime }}\frac{\sqrt{r^{2}+P^{*}}+\frac{\sqrt{P^{*}}A_{k}}{\sqrt{r^{2}+P^{*}}\cdot \sqrt{\alpha_{k-1}^{\prime }}}}{P^{*}+r^{2}+2\sqrt{\frac{P^{*}}{\alpha_{k-1}^{\prime }}}A_{k}}\\ \; & \; & =\frac{r\sqrt{\alpha_{k-1}^{\prime }}}{\sqrt{r^{2}+P^{*}}}\frac{r^{2}+P^{*}+\sqrt{\frac{P^{*}}{\alpha_{k-1}^{\prime }}}A_{k}}{P^{*}+r^{2}+2\sqrt{\frac{P^{\prime }}{\alpha_{k-1}^{\prime }}}A_{k}}\overset{\left(m\right)}{\leq }\frac{r\sqrt{\alpha_{k-1}^{\prime }}}{\sqrt{r^{2}+P^{*}}}, \end{array}$$

where (m) follows from (A13) that $A_{k}\geq 0$. From (A16), we can conclude that

(A17) $$\begin{array}{ccc} & & \sqrt{\alpha_{N}^{\prime }}\leq \frac{r}{\sqrt{r^{2}+P^{*}}}\sqrt{\alpha_{N-1}^{\prime }}\leq \ldots \leq {\left(\frac{r}{\sqrt{r^{2}+P^{*}}}\right)}^{N-1}\sqrt{\alpha_{1}^{\prime }}\\ & & \overset{\left(n\right)}{=}{\left(\frac{r}{\sqrt{r^{2}+P^{*}}}\right)}^{N-1}\sqrt{\frac{\sigma^{2}+\left(1-\rho^{2}\right)P_{2}}{12P^{*}}}, \end{array}$$

where (n) follows from (34). Finally, we bound $P_{e1}$ as follows

(A18) $$\begin{array}{ccc} \; & \; & P_{e1}\leq Pr\left\{\left|\epsilon_{N}^{\prime }\right|>\frac{1}{2\left(\left|W_{1}\right|-1\right)}\right\}\\ \; & \; & \overset{\left(o\right)}{\leq }2Q\left(\frac{1}{2\cdot 2^{NR_{1}}}\cdot \frac{1}{\sqrt{\alpha_{N}^{\prime }}}\right)\\ \; & \; & \overset{\left(p\right)}{\leq }2Q\left(\frac{1}{2}\cdot 2^{-NR_{1}}{\left(\frac{r}{\sqrt{r^{2}+P^{*}}}\right)}^{-N+1}\sqrt{\frac{12P^{*}}{\sigma^{2}+\left(1-\rho^{2}\right)P_{2}}}\right)\\ \; & \; & =2Q\left(\frac{1}{2}\sqrt{\frac{12P^{*}}{\sigma^{2}+\left(1-\rho^{2}\right)P_{2}}}2^{-NR_{1}}{\left(\frac{\sqrt{r^{2}+P^{*}}}{r}\right)}^{N-1}\right)\\ \; & \; & =2Q\left(\frac{1}{2}\sqrt{\frac{12P^{*}}{\sigma^{2}+\left(1-\rho^{2}\right)P_{2}}}2^{-NR_{1}}2^{\left(N-1\right)\log\frac{\sqrt{r^{2}+P^{*}}}{r}}\right)\\ \; & \; & =2Q\left(\frac{1}{2}\sqrt{\frac{12P^{*}}{\sigma^{2}+\left(1-\rho^{2}\right)P_{2}}}2^{-\log\frac{\sqrt{r^{2}+P^{*}}}{r}}2^{-N\left(R_{1}-\log\frac{\sqrt{r^{2}+P^{*}}}{r}\right)}\right), \end{array}$$

where (o) follows from Q(x) and is the tail of the unit Gaussian distribution evaluated at *x*, and (p) follows from (A17) and the fact that Q(x) is decreasing while *x* is increasing. From (A18), we can conclude that if

(A19) $$R_{1}<\log\frac{\sqrt{r^{2}+P^{*}}}{r}\;=\;\frac{1}{2}\log\left(1+\frac{P^{*}}{r^{2}}\right)\overset{\left(q\right)}{\;=\;}\frac{1}{2}\log\left(1+\frac{P_{1}+\rho^{2}P_{2}+2\sqrt{P_{1}P_{2}}\rho }{\left(1-\rho^{2}\right)P_{2}+\sigma^{2}}\right),$$

where (q) follows from (29) and (A15), $P_{e1}\to 0$ as N→∞. The proof of Lemma 1 is completed.
